# Has Ghana's Rotavirus Vaccine Switch Met Programmatic Expectations? An Analysis of National Surveillance Data; 2018–2022

**DOI:** 10.1093/ofid/ofae539

**Published:** 2024-09-27

**Authors:** Michael Rockson Adjei, Justice Ofori Amoah, George Bonsu, Rafiq Okine, Naziru Tanko Mohammed, Kwame Amponsa-Achiano, Franklin Asiedu-Bekoe, Patrick Kuma-Aboagye, Jason Mathiu Mwenda, Martin Peter Grobusch, Sally-Ann Ohene

**Affiliations:** Department of Infectious Diseases, Center of Tropical Medicine and Travel Medicine, Amsterdam University Medical Centers, location AMC, University of Amsterdam, Amsterdam, The Netherlands; Country Office, World Health Organization, Accra, Ghana; Ghana Health Service, District Health Directorate, Effiduase, Ghana; PATH, Accra, Ghana; Headquarters, World Health Organization, Geneva, Switzerland; Headquarters, Ghana Health Service, Accra, Ghana; Headquarters, Ghana Health Service, Accra, Ghana; Headquarters, Ghana Health Service, Accra, Ghana; Headquarters, Ghana Health Service, Accra, Ghana; Regional Office for Africa, World Health Organization, Brazzaville, Republic of Congo; Department of Infectious Diseases, Center of Tropical Medicine and Travel Medicine, Amsterdam University Medical Centers, location AMC, University of Amsterdam, Amsterdam, The Netherlands; Institute of Tropical Medicine, and German Center of Infectious Diseases (DZIF), University of Tübingen, Tübingen, Germany; Institute of Infectious Diseases and Molecular Medicine, University of Cape Town, Cape Town, South Africa; Centre de Recherches Médicales en Lambaréné (CERMEL), Lambaréné, Gabon; Masanga Medical Research Unit, Masanga, Sierra Leone; Country Office, World Health Organization, Accra, Ghana

**Keywords:** childhood immunization, Rotarix, Rotavac, rotavirus, vaccine switch

## Abstract

**Background:**

Ghana introduced a 2-dose schedule rotavirus vaccine, Rotarix, into childhood immunization in 2012 but switched to a 3-dose schedule vaccine, Rotavac, in 2020 on account of programmatic advantages offered by the latter, including lower cost per fully immunized child and lower cold chain volume requirement. The objective of the study was to assess the effect of the vaccine switch on the trends of rotavirus vaccine uptake and health facility outpatient department (OPD) attendance due to diarrhea among children aged 1–11 months.

**Methods:**

A retrospective analysis was conducted on childhood immunization and diarrhea surveillance data for 2018–2022. The uptake of the different rotavirus vaccine products and the proportion of health facility OPD attendance attributed to diarrhea, respectively, were compared between the pre- and postswitch study periods.

**Results:**

The uptake of rotavirus vaccine was sustained following the switch. There were no significant differences in vaccination coverages (rota1, Rotarix coverage [94.3%], vs rota1, Rotavac coverage [95.3%]; *P* = .757; rota2, Rotarix coverage [91.3%], vs rota2, Rotavac coverage [92.7%]; *P* = .789). The proportions of health facility OPD attendance due to diarrhea were comparable (preswitch [12.4%] vs postswitch [12.1%]; *P* = .838).

**Conclusions:**

Ghana's rotavirus vaccine switch yielded expected programmatic benefits without any untoward effects. The trends of vaccine uptake and reduction in diarrhea morbidity were sustained. These experiences and lessons from the rotavirus vaccine switch are vital for potential switches for other vaccines in the current immunization schedule to mitigate the annual vaccine expenditure.

Rotavirus accounts for 35%–60% of severe acute diarrhea in children under 5 years of age in countries without rotavirus vaccine, with the highest attributable percentage in infants [1, 2]. The infection is transmitted directly from person to person, feco-orally, and indirectly through contact with contaminated fomites [1, 2]. Rotavirus remains a leading cause of diarrhea-related illness among children under 5 years, causing nearly 361 000 (81.3%) of the estimated 444 000 global deaths from diarrhea among this age group in 2021 [3]. In Ghana, diarrhea ranks second among the top causes of health facility outpatient department (OPD) attendance for children under 5 years of age [4]. Before the introduction of rotavirus vaccine in the country in 2012, rotavirus accounted for ∼25% of diarrhea-related hospitalizations and deaths in this age group [5–8].

The universal occurrence of rotavirus infections shows that clean water supplies and good hygiene alone are unlikely to have a substantial effect on virus transmission [9]. The World Health Organization (WHO) recommended the introduction of rotavirus vaccine into childhood immunization programs in 2009 [10], and at that time, only 2 vaccine products had been prequalified globally: the monovalent Rotarix vaccine (RV1, GlaxoSmithKline, Rixensart, Belgium; 2-dose series) and the pentavalent RotaTeq vaccine (RV5, Merck, Rahway, NJ, USA; 3-dose series) [11]. In January 2018, a new monovalent rotavirus vaccine product, Rotavac (RV1, Bharat Biotech, Hyderabad, India; 3-dose series), was prequalified by the WHO. As of March 2022, just over half of all countries globally had introduced rotavirus vaccine into their immunization programs [12].

In 2012, Ghana introduced a 2-dose schedule monovalent rotavirus vaccine, Rotarix, into childhood immunization but switched to a different monovalent vaccine product, Rotavac, with a 3-dose schedule in January 2020 [12]. The 2 vaccines have comparable safety and efficacy profiles, but the decision to switch was based on the programmatic advantages offered by the latter. First, the vaccine product Rotavac came in a multidose vial (5 and 10; Ghana opted for the 5-dose vial) and therefore required less cold chain volume to store compared with the first vaccine product, Rotarix, which was packaged as single-dose vials. Secondly, Rotavac was cheaper in terms of the cost per fully immunized child, and potentially reduced governmental annual expenditure on vaccine procurement [12].

Some comparative advantages of Rotavac including being relatively cheaper and requiring lower cold chain volume were immediately verifiable, but vaccine acceptability and effect on diarrhea morbidity were only assessable postintroduction. Although lessons from countries where the vaccine was in use could be inferred, the country-specific (sometimes even regional) setting is critical in determining the outcome of vaccine introduction, hence the need for a context-based evaluation.

The objective of this study was to assess the effect of the vaccine switch on the trends of rotavirus vaccine uptake and health facility OPD attendance due to diarrhea among children aged 1–11 months over a 5-year period (2018–2022). The findings are envisaged to facilitate evaluation of the switch decision and contribute to program strengthening for future vaccine product switches.

## METHODS

### Implementing the Vaccine Switch

Ghana is a low- to middle-income country in Sub-Saharan Africa with 16 administrative regions. The 2021 population and housing census estimated the total population to be 30.8 million at a growth rate of 2.1%, with the proportion of surviving infants constituting ∼4% (1 232 000) [13]. The country's immunization program started in 1978, and as of December 2022, 11 vaccines protecting against 14 vaccine-preventable diseases including coronavirus disease 2019 (COVID-19) had been introduced [14].

In 2018, the National Immunization Technical Advisory Group (NITAG) began discussions with the Ministry of Health (MoH) on evidence-based strategies to sustain the national immunization program as part of the country's preparations to exit Gavi support in 2030 [15]. In 2019, the MoH endorsed the NITAG's recommendation to switch from Rotarix to Rotavac due to the lower price and smaller cold chain volume requirement of the latter [15].

In 2020, the switch implementation commenced with training of health care workers at all levels of the health system, and community engagement to sensitize caregivers and generate demand for uptake [15]. Both rotavirus vaccine products were deployed concurrently, with each eligible child receiving a specific product for all schedules; “mixing and matching” was not encouraged. Despite the impact of the COVID-19 pandemic, the transition was successfully completed by the end of 2020, and all unused doses of the first vaccine product were returned from the subnational levels to the central cold room for disposal [15].

### Study Design, Data Collection, and Analysis

A retrospective analysis was conducted using immunization and diarrhea surveillance data from the District Health Information Management System (DHIMS-2) for 2018–2022.

### Data Collection

Data on rotavirus vaccine uptake, total OPD attendance, and OPD attendance due to diarrhea among children aged 1–11 months were retrieved from DHIMS-2 into Microsoft Excel 16.0 spreadsheet. After checking for completeness, data were imported into Epi Info statistics software (Epi Info, version 7.2.2.16, www.cdc.gov>epiinfo) for analysis.

DHIMS-2 is a free open-source software (https://www.dhis2.org) developed using the District Health Information System (DHIS). It was first introduced in Ghana in the year 2007 and is primarily used for collecting and reporting aggregate data on health service delivery. Data are collected at health facility levels using standard registers, collated onto summary forms, and entered into the DHIMS-2 platform weekly or monthly. The interfase is password-protected and accessible only by authorized health care managers.

### Data Analysis

**Figure 1.**
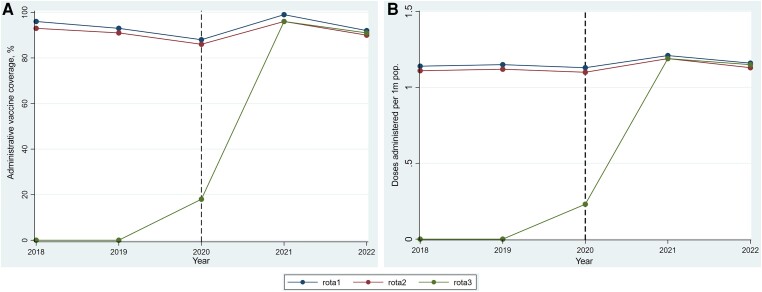
*A*, Rotavirus vaccination coverage by year, Ghana; 2018–2022. *B*, Doses of rotavirus vaccine administered per schedule, Ghana; 2018–2022.

The study period was categorized into 3 phases based on the use of a specific rotavirus vaccine product in the childhood immunization program. Rotarix was used in 2018–2019 (preswitch period); in 2020, both products were administered alongside each other (switch period); and in 2021–2022 (postswitch period), Rotavac was the sole product delivered in the immunization program (Figure 1).

Descriptive statistics were performed. The difference in uptake of rotavirus vaccine products was assessed by comparing the average coverages of the first 2 doses (rota1 and rota2, respectively) between the preswitch and postswitch periods using the chi-square test at a 95% confidence level. Similarly, the proportions of total facility OPD attendance attributed to diarrhea in the preswitch and postswitch periods were compared, adjusting for the approximately 10% decline in diarrhea-related morbidity attributed to improved water, sanitation, and hygiene during the COVID-19 pandemic [16].

## RESULTS

The mean and median data completeness rates over the study period were 93.9% and 92.5%, respectively, ranging from 91.4% for 2018 to 97.8% for 2022, respectively. A total of 14 014 206 doses of rotavirus vaccine were administered over the study period. Approximately 32.3% (4 531 274/14 014 206) were administered in the preswitch period, and 50.1% (7 019 148/14 014 206) in the postswitch period. About 41.4% (5 799 979/14 014 206) were administered as first dose (rota1), 40.3% (5 651 539/14 014 206) as second dose (rota2), and 18.3% (2 562 688/14 014 206) as third dose (rota3). There were no significant differences in uptake of rotavirus vaccine comparing the preswitch and postswitch periods (rota1, Rotarix [94.3%], vs rota1, Rotavac [95.3%]; *P* = .757; rota2, Rotarix [92.9%], vs rota2, Rotavac [91.3%]; *P* = .789). The uptake of rota3 rose from ∼18% in 2020 to at least 90% for 2021 and 2022, respectively (Figure 1).

**Figure 2.**
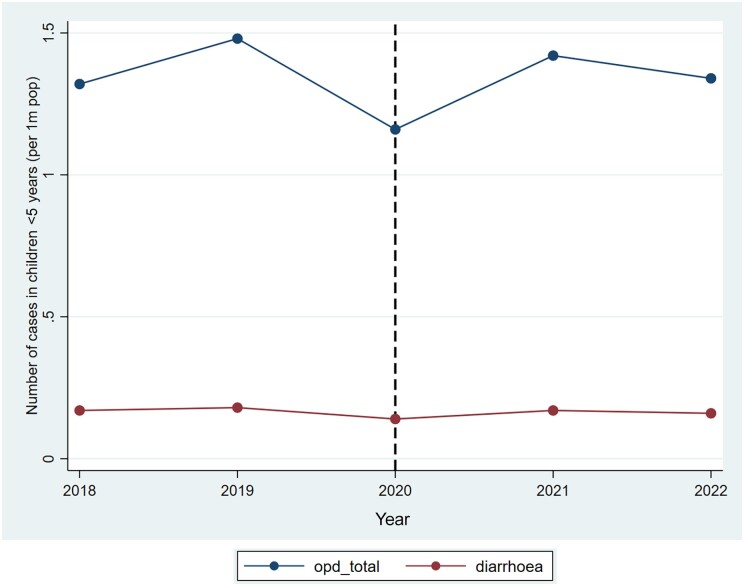
Health facility attendance among children aged 1–11 months, Ghana, 2018–2022.

A total of 6 717 442 health facility OPD attendances were recorded over the study period, and nearly equal proportions, 41.7% (2 798 783/6 717 442) and 41.1% (2 758 215/6 717 442), took place in the preswitch and postswitch periods, respectively. Approximately 12.2% (820 262/6 717 442) of total health facility OPD attendance for the study period was attributed to diarrhea (Figure 2). About 12.4% (346 255/2 798 783) and 12.1% (332 808/2 758 215) of the OPD attendance were attributed to diarrhea in the preswitch and postswitch periods, respectively. There was no significant difference between the proportions of OPD attendance attributed to diarrhea (preswitch vs postswitch: *P* = .838) (Table 1).

**Table 1.** Comparison of Diarrhea Morbidity Among Children Aged 1–11 Months, Ghana, 2018–2022

OPD Attendance	Preswitch Period (P1)	Postswitch		*P* Value (for Comparison of Proportions)	
		Observed (P2a)	Expected (P2b)^a^	P2a vs P1	P2b vs P1
Diarrhea	346 255	332 808	366 125	.838	.838
Total (all conditions)	2 798 783	2 758 215	3 034 340		
Proportion (diarrhea/total, all conditions)	0.124	0.121	0.121		

Abbreviations: COVID-19, coronavirus disease 2019; OPD, outpatient department; WaSH, water, sanitation, and hygiene.

^a^Adjusted for the 9.91% reduction in diarrhea cases/episodes on account of improved WaSH during the COVID-19 pandemic [16].

## DISCUSSION

This study assessed the trends of rotavirus vaccine uptake and the burden of diarrhea disease among children aged 1–11 months over a 5-year period (2018–2022). Vaccine acceptance was sustained over the study period; there was no significant change in rotavirus vaccination coverage following the switch. Of interest, there was no significant difference in the proportion of health facility OPD attendance due to diarrhea, comparing the preswitch and postswitch periods, implying that the vaccine effectiveness of the 2 vaccine products might be comparable [12]. The decline in rotavirus-associated diarrhea among infants following the introduction of rotavirus vaccine in Ghana [6] was sustained after the vaccine switch (Komfo Anokye Teaching Hospital, rotavirus surveillance activity report, 2023, unpublished), which is consistent with the observations reported here.

Based on our findings, it can be inferred that Ghana's rotavirus vaccine product switch yielded expected programmatic objectives. This outcome is a testament of existing collaboration between the national immunization program and health partners in the planning and implementation of new vaccine introductions [17]. Health care workers are the face of health care delivery, and their acceptance of a vaccine tends to mirror that of caregivers and the community [18]. The national immunization program built the capacity of health care workers at all levels of the health system in key areas including demand generation, vaccine management, and vaccine administration (Ghana Health Service, Expanded Programme on Immunization [EPI], training for rotavirus vaccine introduction, 2020, unpublished). This enhanced their capacity to lead demand generation and to respond to concerns of caregivers and communities. Again, capacity building strengthened cold chain management, and this might have contributed to maintenance of vaccine quality.

Newly introduced vaccines require a focused surveillance system within the framework of Integrated Disease Surveillance and Response (IDSR) to monitor safety and impact [19, 20]. The EPI collaborated with other public institutions and leveraged their capacities for the performance of these functions. Following introduction of rotavirus vaccine in Ghana in 2012, the existing rotavirus sentinel surveillance sites were revamped to support monitoring of vaccine impact [19]. The switch to a new product, therefore, did not warrant the establishment of an entirely new system. The Ghana Food and Drugs Authority—the national regulatory authority—continued to monitor the safety of Rotavac through its drug safety monitoring system and provided feedback to the EPI to guide programmatic decisions.

The 3-dose schedule of Rotavac proved to be favorable in terms of its capacity to be integrated into the childhood immunization schedule, given that it aligned with the schedules of oral polio vaccine, pentavalent vaccines (diphtheria-tetanus-pertussis, Hib, and hepatitis B), and pneumococcal conjugate vaccine (PCV), which were administered at 6, 10, and 14 weeks respectively. Again, because the route of vaccine administration was oral, there were no reported caregiver concerns about the additional dose administered for the schedule of Rotavac.

However, the vaccine switch did not come without challenges. The uptake of the third dose of the new vaccine product was spuriously low at some service delivery points in the early stages of the switch due to lapses in recording and documentation (WHO, Ghana—EPI monitoring and supervision report, 2023, unpublished). This might have occurred because of concurrent use of the 2 vaccine products, given the possible transpositional errors that could ensue in managing the recordings for the different schedules. However, the coverage of the third dose rose to par with the first and second doses in the years following the switch.

A limitation of the study was that routine data are often incomplete due to data quality challenges including missing reports. Nevertheless, it is unlikely that this would have a significant effect on the study findings, considering the high data completeness rate (>90%). Further, not all diarrhea episodes included in the study were laboratory confirmed. However, this might not impact the study findings, given that rotavirus is a significant cause of diarrhea among the study population; additionally, proportional OPD attendances due to diarrhea were compared, instead of absolute episode counts, to limit the impact of other causes of diarrhea. Lastly, further studies are required to compare the effectiveness of the 2 vaccine products.

## CONCLUSIONS

This study assessed the outcome of Ghana's switch between rotavirus vaccine products over a 5-year period. The study supports the rationale for the vaccine product switch, given the programmatic benefits of Rotavac and the fact that there was no significant change in trends of rotavirus vaccine uptake or diarrhea-related diseases following the switch.

Although the majority of the implementation challenges were resolved within the first year postswitch, the EPI Programme should enhance timely uptake of the second and third doses by strengthening demand generation and service delivery; should continuously monitor coverage, especially for the third dose; and should institute corrective measures to avert spurious declines. Additionally, given the country's imminent transition from the Gavi program to full self-financing by 2030, the experiences and lessons from the rotavirus vaccine switch are vital for potential switches for other vaccines in the current immunization schedule to mitigate the annual vaccine expenditure.
