# Correction to: Has Ghana's Rotavirus Vaccine Switch Met Programmatic Expectations? An Analysis of National Surveillance Data; 2018–2022

**DOI:** 10.1093/ofid/ofae624

**Published:** 2024-10-16

This is a correction to: Michael Rockson Adjei, Justice Ofori Amoah, George Bonsu, Rafiq Okine, Naziru Tanko Mohammed, Kwame Amponsa-Achiano, Franklin Asiedu-Bekoe, Patrick Kuma-Aboagye, Jason Mathiu Mwenda, Martin Peter Grobusch, Sally-Ann Ohene, Has Ghana's Rotavirus Vaccine Switch Met Programmatic Expectations? An Analysis of National Surveillance Data; 2018–2022, *Open Forum Infectious Diseases*, Volume 11, Issue 10, October 2024, ofae539, https://doi.org/10.1093/ofid/ofae539

In the originally published version of this article, figure legends were inadvertently omitted for figures 1 and 2. This error, for which the publisher apologizes, has been corrected.

